# Dietary Nano-ZnO Is Absorbed via Endocytosis and ZIP Pathways, Upregulates Lipogenesis, and Induces Lipotoxicity in the Intestine of Yellow Catfish

**DOI:** 10.3390/ijms222112047

**Published:** 2021-11-07

**Authors:** Shu-Wei Chen, Wu-Hong Lv, Kun Wu, Guang-Hui Chen, Fang Chen, Chang-Chun Song, Zhi Luo

**Affiliations:** 1 Hubei Hongshan Laboratory, Fishery College, Huazhong Agricultural University, Wuhan 430070, China; chenshuwei@webmail.hzau.edu.cn (S.-W.C.); lvwuhong@webmail.hzau.edu.cn (W.-H.L.); pervcy@webmail.hzau.edu.cn (K.W.); cgh0626@webmail.hzau.edu.cn (G.-H.C.); chenfang95@webmail.hzau.edu.cn (F.C.); songchangchun@webmail.hzau.edu.cn (C.-C.S.); 2 Laboratory for Marine Fisheries Science and Food Production Processes, Qingdao National Laboratory for Marine Science and Technology, Qingdao 266237, China

**Keywords:** nano-ZnO, absorption pathway, lipid metabolism, lipotoxicity, vertebrates

## Abstract

Nano-sized zinc oxide (nano-ZnO) affects lipid deposition, but its absorption patterns and mechanisms affecting lipid metabolism are still unclear. This study was undertaken to investigate the molecular mechanism of nano-ZnO absorption and its effects on lipid metabolism in the intestinal tissues of a widely distributed freshwater teleost yellow catfish *Pelteobagrus fulvidraco*. We found that 100 mg/kg dietary nano-ZnO (H-Zn group) significantly increased intestinal Zn contents. The *zip6* and *zip10* mRNA expression levels were higher in the H-Zn group than those in the control (0 mg/kg nano-ZnO), and *zip4* mRNA abundances were higher in the control than those in the L-Zn (50 mg/kg nano-ZnO) and H-Zn groups. Eps15, *dynamin1*, *dynamin2*, *caveolin1,* and *caveolin2* mRNA expression levels tended to reduce with dietary nano-ZnO addition. Dietary nano-ZnO increased triglyceride (TG) content and the activities of the lipogenic enzymes glucose 6-phosphate dehydrogenase (G6PD), 6-phosphogluconate dehydrogenase (6PGD), and isocitrate dehydrogenase (ICDH), upregulated the mRNA abundances of lipogenic genes *6pgd*, *fatty acid synthase (fas),* and *sterol regulatory element binding protein 1 (srebp1)*, and reduced the mRNA expression of *farnesoid X receptor (fxr)* and small heterodimer partner (*shp*). The SHP protein level in the H-Zn group was lower than that in the control and the L-Zn group markedly. Our in vitro study indicated that the intestinal epithelial cells (IECs) absorbed nano-ZnO via endocytosis, and nano-Zn-induced TG deposition and lipogenesis were partially attributable to the endocytosis of nano-ZnO in IECs. Mechanistically, nano-ZnO-induced TG deposition was closely related to the metal responsive transcription factor 1 (MTF-1)-SHP pathway. Thus, for the first time, we found that the lipogenesis effects of nano-ZnO probably depended on the key gene *shp,* which is potentially regulated by MTF1 and/or FXR. This novel signaling pathway of MTF-1 through SHP may be relevant to explain the toxic effects and lipotoxicity ascribed to dietary nano-ZnO addition.

## 1. Introduction

Nanoparticles (NPs) are defined as small materials with at least one dimension in the 1–100 nm range [1]. Among these NPs, nano-scaled zinc oxide or zinc oxide nanoparticles (nano-ZnO or ZnO NPs) have been used widely in the fields of feed additives, cosmetics, material science, biomedicine, optics, and electronics [1,2]. The widespread use of ZnO NPs has led to their distribution in the environment. The concentration of nano-ZnO was estimated to be 100 µg/L (water) and a few mg/kg (soil) in the UK environment [2]. Since most NPs end up in aquatic environment, there are great concerns towards their aquatic toxicity. At present, several studies have explored the effects of waterborne nano-ZnO exposure on mortality, development, and antioxidant responses in fish [3,4,5]. For example, Zhu et al. [3] found that the 96-h LC50 of nano-ZnO on zebrafish survival was 1.793 mg/L. However, studies involved in the effects of dietary nano-ZnOs addition on fish were scarce [1,6]. In fact, studies suggested that the diet was an important source for mineral absorption [6]. Thus, the exposure to different nanomaterials via oral consumption is an outstanding question, as it is unknown whether dietborne nano-ZnO have chronic effects on the intestine. 

In vertebrates, Zn is mainly absorbed in the intestinal tract and accordingly the intestine is one of the main tissues for nano-ZnO accumulation [7]. However, the absorption pattern and adverse effects of dietborne nano-ZnO exposure are largely unclear. Some studies pointed out that the solubility of nano-ZnO was a key property relevant to its availability and acting effects [7,8]. For example, Tuomela et al. [8] pointed out that the cellular responses to nano-ZnO highly depended on the dissolution of free Zn^2+^ ions, which are absorbed and transported by ZIP transporters. ZIP transporters play important roles in the uptake and transport of Zn from outside the cell into the cytoplasm [9]. On the other hand, nano-ZnO can be absorbed by the cells via the endocytic pathway [10] Thus, it seems that the effects of nano-ZnO are a combination of dissociated free Zn^2+^ ions and nanoparticles. Understanding which pathway is involved in the absorption and effects of nano-ZnO is essential for elucidating the regulatory mechanism of nano-ZnO and for the prevention of potential nanotoxicity. 

In vertebrates, lipids are the main types for energy storage and also have many important physiological functions [11]. However, excess lipid deposition will induce lipotoxicity and be harmful. The intestines are the predominant sites for lipid digestion and absorption and also play important roles in lipid metabolism [11,12]. However, because the intestines are not physiological sites for the lipid storage, excessive lipid accumulation in the intestines is linked with cellular dysfunction. Thus, the regulatory mechanisms in the intestinal lipid uptake and metabolism are extremely interesting from nutritional and pathological perspectives. The lipid metabolism is controlled by many key enzymes and transcription factors. Among these enzymes, fatty acid synthase (FAS), malic enzyme (ME), acetyl-CoA carboxylase (ACC), glucose-6-phosphate dehydrogenase (G6PD), and 6-phosphogluconate dehydrogenase (6PGD) are important lipogenic enzymes and responsible for the regulation of lipogenesis [13,14]. Carnitine palmitoyl transferase I (CPT I) and hormone sensitive lipase (HSL) are two key enzymes involved in lipolysis [14,15]. The intestines also possess a fatty acid transport system, and several proteins, such as fatty acid transport protein (FATP), fatty acid binding protein (FABP), and CD36, mediate the control of fatty acid transport [12,16,17]. In addition, sterol regulatory element-binding protein-1 (SREBP-1), the farnesoid X receptor (FXR), and short heterodimer partner (SHP) are important transcription factors controlling lipid metabolism [18,19,20]. Metal-responsive transcription factor-1 (MTF-1) is the only transcription factor for Zn sensing in vertebrates and constitutes a direct link between Zn and lipid metabolism by targeting genes relevant to lipid metabolism [21,22]. Due to the relevance of intestinal lipid uptake in the development of metabolic disorders, the elucidation of nano-ZnO modulating intestinal lipid levels has emerged as an important target of nutrition research.

Yellow catfish *Pelteobagrus fulvidraco* is an omnivorous freshwater teleost, widely farmed in several Asian countries for their excellent fillet quality and high economic value. The fish species has been considered to be a good model for investigating the molecular and cellular mechanisms underlying the absorption and metabolic effects of minerals since its genome information is known [23], and since it shares similar metabolic pathways and regulatory mechanisms with other vertebrates. In this study, we explored the absorption mechanism of nano-ZnO and its effect on lipid deposition and metabolism in the intestine, which mechanistically provided the insights for the evaluation of Zn nutrition in vertebrates. Our study has uncovered a novel regulatory pathway consisting of MTF-1/SHP, which responds to mineral addition and controls lipid metabolism. Our finding may benefit the development of new management for promoting mineral utilization and for developing prevention strategies for lipotoxicity and lipid metabolism disorder.

## 2. Results

### 2.1. In Vivo Assessment

#### 2.1.1. Zn Accumulation and Absorption in the Intestinal Tissues

**Figure 1:**
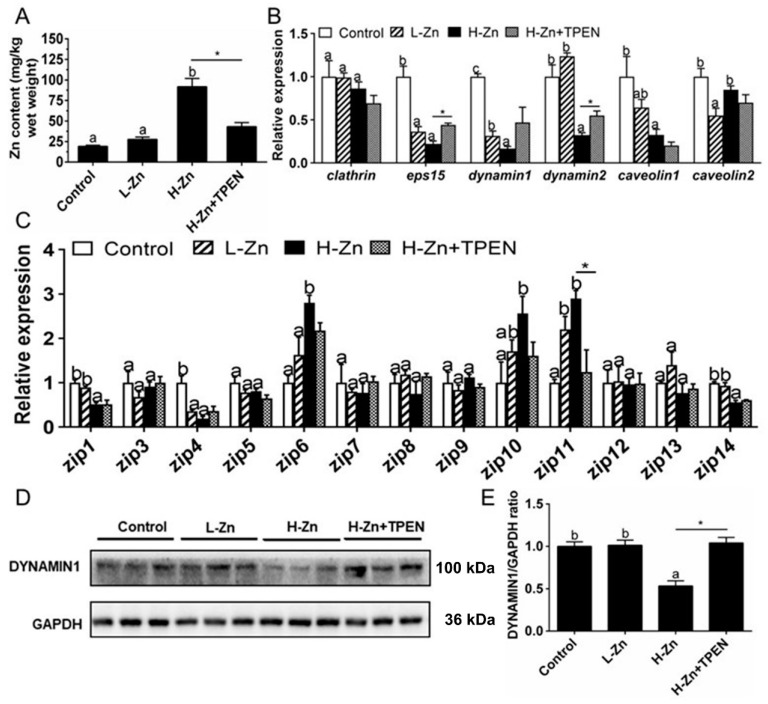
Zn accumulation and transport in the intestines of yellow catfish fed four experimental diets. (**A**) Zn content. (**B**) The mRNA expression of endocytosis genes. (**C**) The mRNA levels of Zn transporters. (**D**) Protein levels of Dynamin1. (**E**) Quantification of Dynamin1 protein level. Values are means ± SEMs, *n* = 3. L-Zn: low dietary Zn; H-Zn: high dietary Zn; H-Zn + TPEN: high dietary Zn + TPEN; Different letters indicate significant differences among three dietary Zn levels (*p* < 0.05). Asterisks (*) indicate significant differences between 100 mg/kg nano-ZnO and the 100 mg/kg nano-ZnO + TPEN group (*p* < 0.05).

Intestinal Zn content was significantly higher in the H-Zn group than in the control and L-Zn group, and TPEN addition alleviated dietary H-Zn-induced Zn accumulation in the intestinal tissues (Figure 1A). For key endocytosis genes, dietary nano-ZnO tended to reduce mRNA levels of *eps15*, *dynamin1,* and *caveolin1* but did not affect *clathrin* mRNA expression (Figure 1B). The *dynamin2* mRNA abundance was lower for the H-Zn group than for the other two groups, and the *caveolin2* mRNA expression was the lowest for the L-Zn group and showed no significant differences between other two groups. The TPEN addition alleviated H-Zn-induced downregulation of *eps15* and *dynamin2* mRNA expression. H-Zn addition also reduced the DYNAMIN1 protein level but the trend was reversed by TPEN addition (Figure 1D,E). Taken together, dietary nano-ZnO addition increased Zn accumulation and influenced the endocytosis pathway.

For the ZIP family proteins, the differences in the mRNA expression of *zip3*, *zip5*, *zip7*, *zip8*, *zip9*, *zip12,* and *zip13* were not statistically significant among the three groups. *zip6* and *zip10* mRNA expression levels were higher, and *zip1* and *zip14* mRNA expression was lower in the H-Zn group than those in the control and L-Zn group. The *zip11* mRNA expression was significantly lower in the control than in other two groups. TPEN addition only alleviated the H-Zn-induced increase in *zip11* mRNA expression but did not significantly influence mRNA expression of the other genes of the ZIP family (Figure 1C). Thus, dietary nano-ZnO addition also influenced the pathway of ZIP-mediated free Zn^2+^ transport. 

#### 2.1.2. Histological Analyses

**Figure 2:**
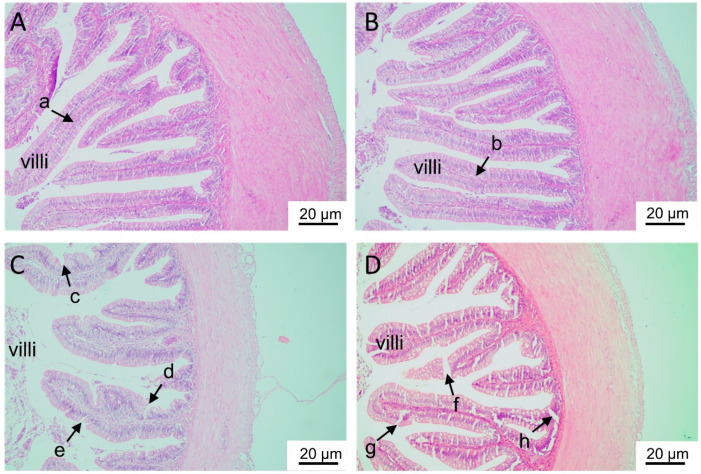
Histological analyses of the intestinal tissues of yellow catfish fed four experimental diets. (**A**) The control. (**B**) 50 mg/kg nano-ZnO group. (**C**) 100 mg/kg nano-ZnO group. (**D**) 100 mg/kg nano-ZnO + TPEN group. In (**A**,**B**), the intestinal villi were complete, full, and intact (arrows a and b). In (**C**,**D**), the villi showed injury, notching and vacuoles (arrows c–h).

H&E staining showed that the intestinal villi were complete and intact in the control and L-Zn groups but showed injury, notching, and vacuoles in the H-Zn and H-Zn + TPEN groups (Figure 2A–D).

#### 2.1.3. TG Content, Activities, and Expression of Enzymes and Genes Involved in Lipid Metabolism

**Figure 3:**
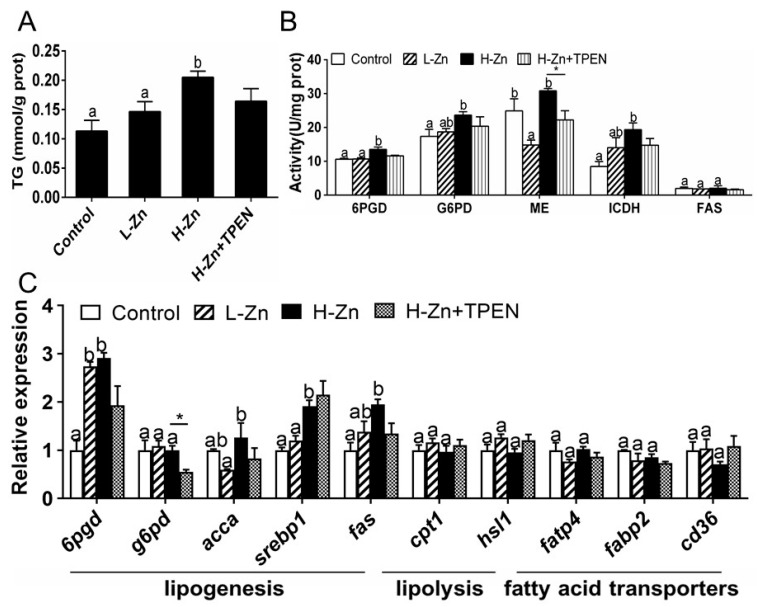
Triglyceride (TG), enzyme activities, and gene expression of the intestinal tissues of yellow catfish fed four experimental diets. (**A**) TG content. (**B**) Enzyme activities. (**C**) The mRNA expression of lipid metabolism-related genes. L-Zn: low dietary Zn; H-Zn: high dietary Zn; H-Zn + TPEN: high dietary Zn + TPEN. Different letters indicate significant differences among three dietary Zn levels (*p* < 0.05). Asterisks (*) indicate significant differences between the 100 mg/kg nano-ZnO group and the 100 mg/kg nano-ZnO + TPEN group (*p* < 0.05). For each value, three biological replicates are undertaken.

Dietary nano-ZnO addition increased the TG contents of the intestine tissues. TPEN addition tended to reduce H-Zn-induced TG content, but the differences were not significant (Figure 3A). The activities of 6PGD, G6PD, and ICDH were the highest for yellow catfish fed the H-Zn diet and showed no marked discrepancies between other two groups (Figure 3B). The ME activity was lowest in the L-Zn group and showed no significant differences between the other two groups. Dietary nano-ZnO addition did not significantly influence FAS activity. Compared to the control, nano-ZnO addition increased the mRNA expression of three lipogenic genes (*6pgd*, *fas,* and *srebp1*) but did not influence the mRNA expression of *g6pd*, *cpt1*, *hsl1*, fatp4, *fabp2,* and *cd36*. *acca* mRNA expression was highest for fish fed the H-Zn diet and showed no marked discrepancies between the other two groups (Figure 3C). TPEN + H-Zn diet reduced *g6pd* mRNA expression compared to the H-Zn group. Altogether, these results indicated that dietary nano-ZnO increased the TG deposition and upregulated lipogenesis, and these effects were partially attributable to free Zn^2+^ dissociated from nano-ZnO.

**Figure 4:**
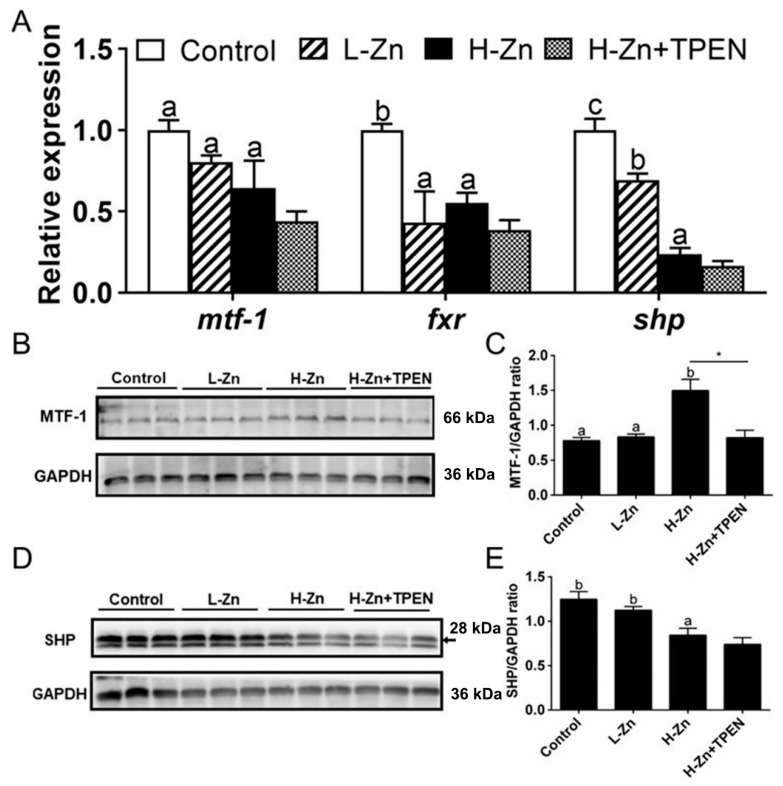
Effects of nano-sized zinc oxide on MTF-1-SHP pathway genes. (**A**) The mRNA levels of MTF-1-SHP pathway genes. (**B**) MTF-1 protein level. (**C**) Quantification of the MTF-1 protein level. (**D**) Protein levels of SHP. (**E**) Quantification of SHP protein level. L-Zn: low dietary Zn; H-Zn: high dietary Zn; H-Zn + TPEN: high dietary Zn + TPEN. Different letters indicate the significant differences among the three dietary Zn levels (*p* < 0.05). Asterisks (*) indicate significant differences between 100 mg/kg nano-ZnO and the 100 mg/kg nano-ZnO + TPEN group (*p* < 0.05). For each value, three biological replicates are undertaken.

Dietary nano-ZnO tended to reduce the mRNA expression of *mtf-1*, *fxr,* and *shp*, but TPEN addition did not influence H-Zn-induced changes of their mRNA expression (Figure 4A). MTF-1 protein level was higher in the H-Zn group than in the other two groups, and TPEN alleviated H-Zn-induced increment of the MTF-1 protein level (Figure 4B,C). Nano-ZnO addition tended to reduce the SHP protein level, and TPEN did not significantly influence H-Zn-induced changes of the SHP protein level (Figure 4D,E). 

### 2.2. In Vitro Assessment

#### 2.2.1. IECs Absorbed Nano-ZnO via Endocytosis Pathways

**Figure 5:**
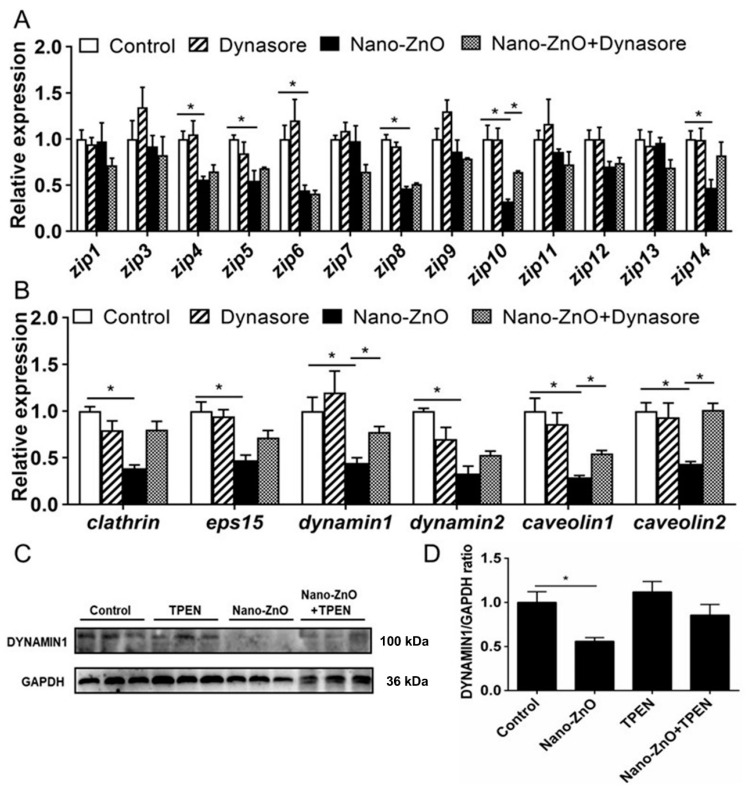
Effects of Nano-ZnO and Dynasore incubation on expression of genes and proteins involved in Zn absorption and transport of yellow catfish IECs. (**A**) The mRNA expression of Zn transport protein. (**B**) The mRNA expression of endocytosis genes. (**C**) Dynamin1 protein levels. (**D**) Quantification of Dynamin1 protein level. Different letters or asterisks (*) indicate the significant differences between the two groups (*p* < 0.05). For each value, three biological replicates are undertaken.

Dynasore preincubation alleviated nano-ZnO-induced reduction in mRNA expression of *zip10*, *dynamin1*, *caveolin1,* and *caveolin2* (Figure 5A,B) and alleviated nano-ZnO-induced reduction in the DYNAMIN1 protein level (Figure 5C,D). Taken together, these results indicated that endocytosis mediated dietary nano-ZnO absorption and transport in the IECs.

#### 2.2.2. Nano-ZnO-Induced TG Deposition and Lipogenesis Was Partially Attributable to the Endocytosis of Nano-ZnO in IECs

**Figure 6:**
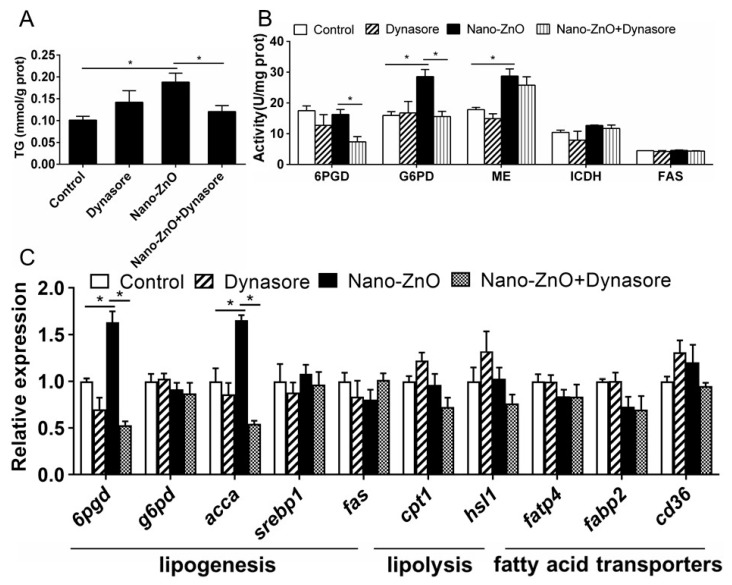
Effects of Nano-ZnO and Dynasore incubation on TG contents, enzymatic activities, and gene expression of yellow catfish IECs. (**A**) TG content. (**B**) Enzyme activities. (**C**) The mRNA expression of lipid metabolism genes. Asterisks (*) indicate the significant differences between the two treatments (*p* < 0.05). For each value, three biological replicates are undertaken.

Dynasore incubation did not significantly affect TG content but alleviated nano-ZnO-induced increase in TG contents (Figure 6A). Dynasore preincubation did not significantly influence nano-ZnO-induced changes in the activities of ME, ICDH, 6PGD, G6PD, and FAS (Figure 6B). Dynasore preincubation alleviated nano-ZnO-induced increase in *6pgd* and *acca* mRNA expression but did not significantly affect the mRNA expression of lipogenic genes (*g6pd*, *srebp1,* and *fas*), lipolytic genes (*cpt1* and *hsl1*), and fatty acid transporters *fatp4*, *fabp2,* and *cd36* (Figure 6C). These results indicated that nano-Zn-induced TG deposition and lipogenesis was partially attributable to the endocytosis of nano-ZnO in IECs.

#### 2.2.3. Nano-ZnO-Induced TG Deposition was Closely Related to the MTF-1-SHP Pathway

**Figure 7:**
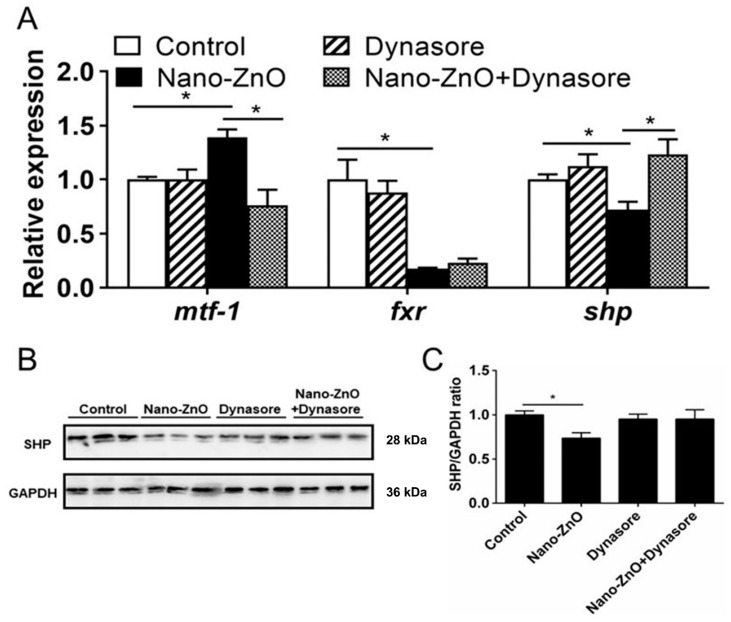
Nano-ZnO-induced triglyceride (TG) deposition was closely related to the MTF-1-SHP pathway with or without Dynasore incubation. (**A**) The mRNA expression of MTF-1-SHP pathway genes. (**B**) Protein levels of SHP. (**C**) Quantification of SHP protein. Asterisks (*) indicate the significant differences between the two groups (*p <* 0.05). For each value, three biological replicates are undertaken.

Dynasore preincubation alleviated nano-ZnO-induced upregulation of *mtf-1* mRNA expression and alleviated nano-ZnO-induced downregulation of *shp* mRNA expression (Figure 7A) but did not significantly influence nano-ZnO-induced reduction in the SHP protein level (Figure 7B,C). These results indicated that the nano-ZnO-induced TG deposition was closely related to the MTF-1-SHP pathway.

## 3. Discussion

The intestinal tract is the main organ for absorption and metabolism of dietborne nutrients. However, studies involved in the absorption and metabolism of dietborne nano-ZnO in the intestine tissues were very scarce. Here, for the first time, we found that the intestine and IECs could absorb Zn via the dissociation of Zn^2+^ ions from nano-ZnO and via the endocytosis of nano-ZnO. Moreover, we found that nano-ZnO increased lipid deposition and lipotoxicity via the upregulation of lipogenesis. Moreover, we identified a novel regulatory pathway consisting of MTF-1/SHP, which can respond to nanoparticle addition and in turn regulate lipid deposition and metabolism.

Our study indicated that high dietary nano-ZnO addition increased intestinal Zn content, in agreement with other studies using nano-Zn [12] and an inorganic Zn source [24]. To explore the mechanism of Zn-induced changes in Zn content, several genes and proteins related to endocytosis were investigated. The high uptake rate of nanoparticles may be attributed to multiple absorption pathways. At first, studies suggested that endocytosis pathways were involved in the internalization of nanoparticles into cells [10]. Clathrin- and caveolin-mediated endocytosis represent the most well studied and major pathways of internalization for the entry of nanoparticles [12]. Our study suggested that dietary nano-ZnO reduced the mRNA levels of *eps15*, *dynamin1*, *dynamin2,* and *caveolin1* and also reduced the DYNAMIN1 protein level. Dynamin1 and dynamin 2 are two GTPase enzymes that play vital roles in the clathrin-dependent endocytosis and other vesicular trafficking processes [25,26]. Epidermal growth factor receptor pathway substrate clone 15 (Eps15) is an adaptor protein that is involved in endocytosis through its interaction with the clathrin adaptor AP-2 at the plasma membrane [27]. Caveolin 1 belongs to the member of caveolin proteins which mediate caveolin1-associated endocytosis [28]. Their reduction in the expression level by nano-ZnO indicated the reduction in clathrin- and caveolin-dependent endocytosis of nano-ZnO, and thus provided a protective mechanism against nano-ZnO uptake. Dynasore is a strong inhibitor for the dynamin-dependent endocytosis [25]. Our in vitro study indicated that dynasore preincubation alleviated nano-ZnO-induced reduction in mRNA expression of *zip10*, *dynamin1*, *caveolin1,* and *caveolin2* and alleviated nano-ZnO-induced reduction in the DYNAMIN1 protein level. These results further demonstrated that clathrin- and caveolin1-dependent endocytosis mediated the absorption and transport of dietary nano-ZnO in the intestine and IECs. Second, we found that high dietary nano-ZnO addition tended to reduce mRNA levels of *zip1*, *zip4,* and *zip14*. The ZIP family plays prominent roles in Zn influx into cytosols [9,24]. Thus, the reduction in their mRNA levels will downregulate excessive Zn transport into the cytosols and help to maintain Zn homeostasis, as suggested by Chen et al. [24]. However, we found that high dietary nano-ZnO addition increased the mRNA levels of *zip6*, *zip10,* and *zip11*. These differences, along with peculiarities in regulation, might have important implications for the roles of these proteins in cellular metal homeostasis, as pointed out by Jenkitkasemwong et al. [29]. Ho et al. [30] suggested that the tissue expression among the Zn transporters were very different. The mechanism underlying these differences requires further study. Generally, our study indicated that dietary nano-ZnO addition influenced the pathway of ZIP-mediated free Zn^2+^ transport. Studies have pointed out that nano-Zn can be dissolved in the digestive tract and release free Zn^2+^, which thereby activates the ZIP transport system to help high efficiency absorption of nano ions [1]. The continuous dissolution of nano-ZnOs in the intestine would provide highly bioavailable free Zn^2+^ [1]. Taken together, intracellular Zn may come from both released Zn^2+^ through the ZIP pathway and nano-ZnO particles through endocytosis system. The collaboration of the endocytosis system and ZIP pathway guaranteed high absorption efficiency of nano-ZnO and maintained cellular Zn at a suitable level. 

Studies suggested that nano-ZnO induced the disorder of lipid metabolism and hyperlipidemia [31]. However, the relevant mechanism remained unclear. In the present study, dietary nano-ZnO addition increased the TG contents of the intestine tissues. Similarly, Ling et al. [12] reported that dietary nano-Zn increased the Zn and TG accumulation in the intestine of yellow catfish. These indicated that nano-ZnO and nano-Zn possessed similar properties in inducing intestinal TG deposition. To explore the effect of dietary nano-ZnO on lipid metabolism, we measured enzyme activities and the mRNA expression of metabolism-related genes. We found that high dietary nano-ZnO addition tended to increase the activities of 6PGD, G6PD, and ICDH and upregulated the mRNA abundances of *6pgd*, *acca*, *srebp1,* and *fas*. 6PGD, G6PD, ICDH, ACCA, FAS, and SREBP1 are key enzymes and genes mediating the lipogenesis [22,32]. Their increase in activities and/or mRNA expression indicated that nano-ZnO upregulated lipogenesis. Similarly, Esmaeillou et al. [33] pointed out that oral administration of ZnO nanoparticle suspension increased the serum triglyceride levels. Wang et al. [1] found that nano-ZnO significantly increased the serum TG content of mice. Ling et al. [12] reported that nano-Zn increased intestinal TG accumulation by upregulating lipogenesis. In addition, our study indicated that dietary nano-ZnO addition did not influence mRNA expression of lipolytic genes (such as *cpt1* and *hsl1*) and fatty acid transport (*fatp4*, *fabp2,* and *cd36*), indicating these processes did not mediate the nano-ZnO-induced changes in TG content. Our study indicated that the TPEN addition tended to decrease H-Zn-induced TG content and *g6pd* mRNA expression. Thus, these results indicated that dietary nano-ZnO addition increased TG deposition and upregulated lipogenesis, and these effects were partially attributable to free Zn^2+^ dissociated from nano-ZnO. Our in vitro study indicated that Dynasore alleviated nano-ZnO-induced increase in TG contents, activities of 6PGD and G6PD, and mRNA expression of *6pgd* and *acca*. These results indicated that TG deposition and lipogenesis induced by nano-ZnO were partially attributable to the endocytosis of nano-ZnO in the IECs.

Investigating the effects of nanoparticles on the signaling pathways and elucidating the mechanisms that regulate these processes will increase our understanding into the effects of nanoparticles [15]. Here, nano-ZnO addition tended to reduce the mRNA expression of *mtf-1*, but the MTF-1 protein level was higher in the H-Zn group than in the other two groups. MTF-1 is the only transcription factor for Zn sensing in the vertebrates and regulates the expression of genes involved in Zn metabolism [24]. Ling et al. [12] found that nano-Zn addition increased intestinal *mtf-1* mRNA and protein level. Here, changes in *mtf-1* mRNA expression were not accompanied by parallel changes in protein level. These discrepancies may be because *mtf-1* mRNA is not stable and degrades rapidly; thus, protein levels and mRNA levels may not change in parallel. In the present study, nano-ZnO addition tended to reduce mRNA expression of *fxr* and *shp* and the SHP protein level. FXR and SHP play important roles in regulating lipid metabolism [18,20]. Their variations in the expression indicated that FXR and SHP mediated nano-ZnO-induced changes of lipid metabolism. In fact, studies suggest that FXR and SHP play a role in alleviating lipid accumulation [18,20]. We also observed the parallel changes in FXR and SHP expression after nano-ZnO feeding. Other studies indicated that FXR was an upstream regulator of SHP [34], and that activation of FXR increased SHP levels [35]. Studies also suggested an interactive role between the SHP and MTF-1, and SHP directly repressed the MTF-1 expression at the transcriptional levels [21]. The induction of MTF-1 expression by Zn inhibits the SHP expression by binding to the promoter of SHP and reducing its promoter activity [21]. Thus, it is postulated that MTF-1 plays a predominant role in the inhibition of SHP. Our study indicated that TPEN alleviated H-Zn-induced the increment of the MTF-1 protein level but did not influence H-Zn-induced changes of the mRNA expression of *mtf-1*, *fxr,* and *shp* and the SHP protein level. As mentioned above, TPEN is a chelator of free Zn^2+^ ions. These results indicated that H-Zn-induced changes of the mRNA expression of *mtf-1*, *fxr,* and *shp* and the SHP protein level are not attributable to free Zn^2+^ ion dissociated from nano-ZnO. Our in vitro study indicated that dynasore preincubation alleviated nano-ZnO-induced upregulation of *mtf-1* mRNA expression and alleviated nano-ZnO-induced downregulation of *shp* mRNA expression but did not significantly influence nano-ZnO-induced reduction in the SHP protein level. These results indicated that the nano-ZnO-induced TG deposition was closely related to the endocytosis of nano-ZnO and to the MTF-1-SHP pathway. Therefore, SHP as the key lipolytic factor and its suppression may cause lipid accumulation under nano-ZnO treatment. 

## 4. Conclusions

**Figure 8:**
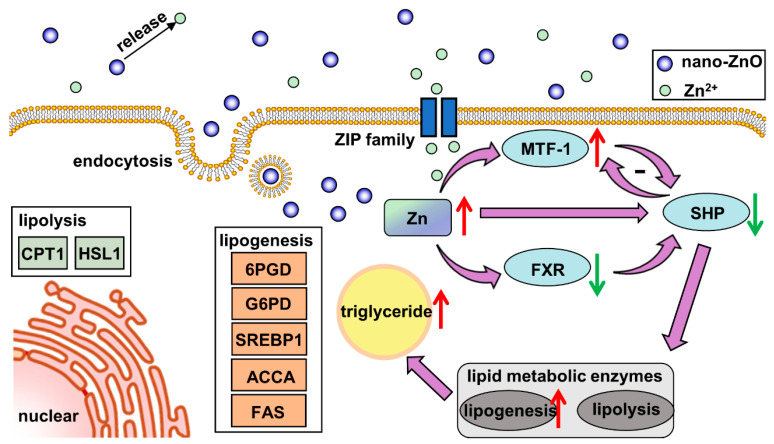
Model diagram of the nano-sized ZnO absorption and its effects on lipid metabolism in yellow catfish.

In our study, dietary nano-ZnO increased the Zn and TG contents and promoted lipogenesis in the intestine. The high uptake rate of nano-ZnO may be attributed to multiple absorption pathways. The nanoparticles were absorbed into intestinal epithelial cells via the endocytosis pathway, while Zn^2+^ released from nano-ZnO was absorpted by the ZIP system. The lipogenesis effects of nano-ZnO probably depended on the key gene SHP, which was regulated by MTF1 and/or FXR (Figure 8). We have provided evidence that a novel regulatory pathway consisting of MTF-1/SHP is critical to the regulation of nano-ZnO-induced lipid deposition. This work provides new evidence for understanding the mechanisms associated with the lipotoxic effects ascribed to dietary nano-ZnO. However, considering that the characteristics of nano-ZnO are influenced by its size and morphology, we should remain cautious of extrapolating the present study to other nanoparticles.

## 5. Materials and Methods

### 5.1. In Vivo Assessment

#### 5.1.1. Fish Culture and Sampling

The diet formulations were presented in Supplementary Table S1. Briefly, 288 yellow catfish (body weight: 8.4 ± 0.4 g per fish, two-month old) were transferred to 12 fiberglass tanks (300 L water), 24 fish per tank. Four experimental diets were formulated, each containing 0 mg/kg (the control), 10 mg/kg (L-Zn group), 100 mg/kg (H-Zn group), and 100 mg/kg (H-Zn + TPEN group) ZnO nanoparticles (nano-ZnO) (average size in one dimension: 50 nm, ≥99% in purity; Sigma-Aldrich company, St. Louis, MO, USA), and 10 mg/kg TPEN was added in the final experimental diets (Supplementary Table S1). Dietary Zn levels were designed based on our previous study [36]. Each diet was randomly assigned to three tanks, with 12 tanks for the experiment. The experiment continued for 10 weeks. 

At the end of the feeding experiment, 24 h after the last feeding, all the yellow catfish were euthanized (MS-222 at 100 mg/L). They were dissected and the anterior intestines were sampled. The intestinal contents were scraped off gently and rinsed with neutral phosphate buffer solution (PBS), and the samples were analyzed for triglyceride (TG) content, enzyme activity, mRNA, and protein level. For the assays of enzyme activities and mRNA expression, the intestine samples were removed using sterile forceps, frozen quickly in liquid N_2_, and stored at −80 °C for further analysis. The remaining samples were stored at −80 °C for the determination of Zn content.

#### 5.1.2. Zn and TG Contents

The Zn contents in the intestine and diets were measured using the inductively coupled plasma atomic emission spectrometry (ICP-AES, MA, SUA) [37]. TG and protein contents were analyzed via the commercial kit from the Nanjing Jian Cheng Bioengineering Institute (Nanjing, China).

#### 5.1.3. Histological Observation

Histological observations were conducted via light microscopy, according to the methods described in our previous publications [22]. 

#### 5.1.4. Enzymatic Activity and Realtime Quantitative PCR (Q-PCR)

Enzyme activities of FAS, G6PD, 6PGD, ICDH, and ME were analyzed as described in our previous study [37]. One unit of enzymatic activity was defined as 1 μM of the substrate converted to the final product per minute at 28 °C and was expressed as mU per mg soluble protein. 

Q-PCR assays were performed with Sybr Green (Millipore, Sigma, MO, USA). We selected the two most stable genes (β-actin and *rpl7*) from eight housekeeping genes (*b2m*, *gapdh*, *rpl7*, β-*actin*, *hprt*, *elfa*, *tbp,* and *tuba*) according to the geNorm software (Center for Medical Genetics, Ghent, Belgium; https://genorm.cmgg.be/, accessed on 21 September 2020) [38]. The 2^−ΔΔCt^ method was used to calculate the fold-change of the relative mRNA expression levels. The primers for Q-PCR are given in Supplementary Table S2.

#### 5.1.5. Western Blot (WB) Analysis

The WB analysis followed the protocols described in previous reports [22]. Briefly, tissue lysates were prepared with a RIPA buffer (Thermo Fisher Scientific, Waltham, MA, USA). The proteins (40 μg from each sample) were then separated on the 12% SDS-polyacrylamide gel, transferred to the PVDF membranes, and then blocked with 8% (*w/v*) skimmed milk in the Tris-buffered saline with a Tween 20 (TBST) buffer for 1 h. The membranes were washed thrice with the TBST buffer for 10 min each, followed by incubation with specific primary antibodies. The antibodies included: rabbit anti-GAPDH (10494-1-AP, Proteintech, Wuhan, China), rabbit anti-MTF1 (25383-1-AP, Proteintech), rabbit anti-SHP (ab96605, Abcam), and rabbit anti-DYNAMIN (18205-1-AP, Proteintech). We visualized the protein bands with enhanced chemiluminescent (ECL) and quantified them with Image-Pro Plus 6.0 software (Media Cybernetics, Rockville, MD, USA).

### 5.2. In Vitro Assessment

#### 5.2.1. Enterocytes Culture and Treatments

Intestinal epithelial cells (IECs) were isolated from the intestine tracts of yellow catfish based on the methods of our previous study [37]. The nano-ZnO was dissolved in ultra-pure water at 10 μg/mL of stock concentrations and then sterilized. Before each use, the nano-ZnO solution was treated with an ultrasonic cleaner for 20 min. Two pathway inhibitors, such as TPEN (Zn^2+^ chelator, Sigma, MO, USA) and Dynasore (DYNAMIN inhibitor, Sigma, MO, USA), were used. Five treatments were designed as follows: the control, 20μg/mL nano-ZnO (nano-ZnO group), 2 μM TPEN (TPEN group), 20 μg/mL nano-ZnO + 2 μM TPEN (nano-ZnO + TPEN group), 2 μM Dynasore (Dynasore group), and 20 μg/mL nano-ZnO + 2 μM Dynasore (nano-ZnO + Dynasore group). The concentrations of Zn and the corresponding inhibitors were selected according to our and other studies [39,40]. Each treatment had triplicate groups. The cells were incubated for 24 h and collected for subsequent analysis. 

#### 5.2.2. TG Content, and Enzymatic Activity

The TG content was measured by the commercial kit from Nanjing Jian Cheng Bioengineering Institute (Nanjing, China). Enzymatic activity was measured as described above.

#### 5.2.3. Q-PCR and Western Blot Analysis

The Q-PCR for the in vitro experiment was conducted based on the methods mentioned above, and β-*actin* and *rpl7* were used as the control genes according to the geNorm software (https://genorm.cmgg.be/, accessed on 9 September 2021) [38]. Supplementary Table S2 lists the specific PCR primers. We performed the WB analysis based on the protocols mentioned above, and the antibodies included: rabbit anti-GAPDH (10494-1-AP, Proteintech), rabbit anti-SHP (ab96605, Abcam), and rabbit anti-DYNAMIN (18205-1-AP, Proteintech)

### 5.3. Statistical Analysis

Statistical analysis was conducted with the SPSS 19.0 software19 (IBM Corp., Armonk, NY, USA). The experimental data were analyzed with one-way ANOVA with the Duncan multiple range test among the treatments (≥three treatments). The Student’s *t*-test was used to compare the difference between two groups. The significance level was set at *p* < 0.05.

## Data Availability

The data presented in this study are available on request from the corresponding author.

## Supplementary Materials

The following are available online at https://www.mdpi.com/article/10.3390/ijms222112047/s1.
