# Supplementary material for: Dietary Nano-ZnO Is Absorbed via Endocytosis and ZIP Pathways, Upregulates Lipogenesis, and Induces Lipotoxicity in the Intestine of Yellow Catfish
Source: Int J Mol Sci. 2021 Nov 7;22(21):12047. doi: 10.3390/ijms222112047 (PMC8584588; doi:10.3390/ijms222112047)
Supplement: Supplementary file 1 [file ijms-22-12047-s001.zip › ijms-1412152-supplementary.pdf]

Table S1. Feed formulation and proximate analysis of experimental diets.

Ingredients (g kg^{-1})	Control	Low ZnO NP	High ZnO NP	High ZnO NP+TPEN
Casein	360	360	360	360
Wheat flour	250	250	250	250
White fish meal	50	50	50	50
Fish oil	25	25	25	25
Soybean oil	25	25	25	25
Ascorbyl-2-polyphosphate	10	10	10	10
NaCl	10	10	10	10
$Ca(H_2PO_4)_2 \cdot H_2O$	10	10	10	10
Vitamin premix	5	5	5	5
Mineral premix (Zn-free)	5	5	5	5
Betaine	10	10	10	10
Gelatin	30	30	30	30
Cellulose	215	214.99	214.9	214.89
ZnO NP (mg)	0	10	100	100
TPEN (mg)	0	0	0	10
Proximate analysis (percentage of dry matter basis)				
Moisture content (%)	9.31	9.00	8.84	8.65
Crude protein (%)	43.36	42.53	42.95	42.51
Ash (%)	2.82	2.79	2.79	2.84
Lipid (%)	7.82	7.79	7.96	7.99
Zn (mg/kg)	28.47	35.87	111.08	111.94

Supplemental Table S2. Primers used for real-time quantitative PCR analysis

Genes	Forward primer (5'-3')	Reverse primer (5'- 3')	Accession no.
ZIP1	GACAGCACGGACA GAAGGAA	TGAAGTGCGCAGAAG GAGTT	MK448206
ZIP3	CAGTCAGCCTCATG ATCCCC	TCGTGTTTGTCCTCCA GCTC	MK448207
ZIP4	CATTCATAACTTCGC AGACGG	CCAGAAAGCAACCCC AG ATT	KY652752
ZIP5	GTCGTGGTGCATGG ATCTTC	AGGTGAAGTGATCTG GGGTG	KY652753

ZIP6	CATCCCCACACTCACACACA	CATGGCAGAACACAGCAACC	MK448208
ZIP7	AGTGGAGTTGTGGATGCAGG	CGTTTCCGTGGTGAGAGTGA	MK448209
ZIP8	TTCAGCATCTCAAGCAGCGA	TCACACACGATGGCAATGGA	MK448210
ZIP9	AGCAGCTTCTACCTCCCAGA	AGCGCCTCTTTACTGCTCTG	MK448211
ZIP10	TAAACAGGCGCTCAGATGCA	GTGGCCGTGCTTCTTTGTTT	MK448212
ZIP11	GAAGGCCTTGCAGTGAGTCT	CCAGCTTCCCATTGCCATTG	MK448213
ZIP12	GTCGCTTCATCTTCCGTTCC	TGTCCTGTTGTGCTCCTCAT	XM_027169508.1
ZIP13	CGAGCGGCTATCTGAACCTT	ACTCCAGCGATCAAACCCAG	MK448215
ZIP14	ATCAGTGGCCATCCTGTGTG	GTTTCCTGCCAGAATCCCGA	MK448216
Clathrin	ACCTGGTCACCCAGAACTTG	ATGCCATGTTCATTCCCATT	XM_027142873.1
Eps15	ATGTCCTCAGCCAGCACTCT	TGGGATGACTCCTCCTTTTG	XM_027166552.1
Dynamin 1	TGCAGCTCATCACCAAGTTC	GATGGCTGTGAGCACGTCTA	XM_027145569.1
Dynamin 2	ATGCCAAGGACTTCATCCAC	ACCGATGATGCTGAGAGCTT	XM_027176505.1
Caveolin 1	GTGGACTTCGAGGACGTGAT	TAGACCTGGCTGATGCAGTG	XM_027160262
Caveolin 2	GAGACATTCACGCGCACTTA	GCGAAAACAAGGCCTGATAC	XM_027160228.1
6PGD	GCTCTGATGTGGCGAGG TGG	CGTAGAAGGACAGTGCAGTGG	JX992745
G6PD	CAGGAATGAACGCTGGG ATG	TCTGCTACGGTAGGTCAGGTCC	JX992744
ACCA	GGGGTTTTCACGCTGCT TC	GGTTCTGATTGGGTCGTCCTG	JX992746
FAS	AACTAAAGGCTGCTGGT TGCTA	CACCTTCCCGTCACAAACCTC	JN579124
CPT1	ATTTGAAGAAGCACCCAGAGTATGT	CCCTTTTATGGACGGAGACAGA	JQ074177
ATGL	AGAGAGACCTGCCTGAACAC	CAGGAGGGAACAGACCACAA	KF614123.1
HSL1	GGACTGCCACCTGTACATCT	CGCGTCCTTAGTCTCTTTGC	KJ588764.1
FATP4	TGCCCCTCACATAGTTGCT G	CACTTCCTCGAACATCC CTCAT	MG637279
FABP2	CTATGAAGGTGTGGAGGCCA	TTTAAATGGTCACCGCGGTC	XM_027147559.1
CD36	TGACCTCACTGAAC	TGGTGGAAACTTTGCT	XM_02716078

	ATCGCT	GACG	3.1
SREBP1	CTGGGTCATCGCTT CTTT GTG	TCCTTCGTTGGAGCTT TTGTCT	JX992742
FXR	CCCATTTAGGCAGG TTCAAA	TCCTTGCACTTTCGGA GTCT	XM_02716201 8.1
SHP	CGCACAAGAGCAC GTAACAT	CAAGCCTCCAGAGTTT GTCC	XM_02716173 8.1
MTF-1	CGAGTTGATGTTGC AGAGCC	GAGGTATGGAGGAAA GAAGGGA	XM_02714891 7.1
β-actin	GGACTCTGGTGATG G TGTGA	CTGTAGCCTCTCTC GGTCAG	EU161066
RPL7	GGCAAATGTACAGG AGCGAG	GCCTTGTTGAGCTT GACGAA	KP938522
HPRT	ATGCTTCTGACCTG G AACGT	TTGCGGTTCAGTGC TTTGAT	KP938523
TUBA	TCAAAGCTGGAGTT C TCGGT	AATGGCCTCGTTAT CCACCA	KP938526
B2M	GCTGATCTGCCATG T GAGTG	TGTCTGACACTGCA GCTGTA	KP938520
UBCE	TCAAGAAGAGCCA G TGGAGG	TAGGGGTAGTCGA TGGGGAA	KP938524
GAPDH	TTTCAGCGAGAGAG ACCCAG	ATGACTCTCTTGGC ACCTCC	KP938521
18SrRN A	AGCTCGTAGTTGGA T CTCGG	CGGGTATTCAGGC GAGTTTG	KP938527
ELFA	GTCTGGAGATGCTG C CATTG	AGCCTTCTTCTCAA CGCTCT	KU886307
